# Knowledge and Attitude Towards Cervical Cancer Screening Among Female Students in Allied Health Colleges in Shinyanga Region

**DOI:** 10.24248/eahrj.v8i1.750

**Published:** 2024-03-28

**Authors:** Zephania Pascal Msunza, Anna Tengia Kessy, Saidah Mohamed Bakar

**Affiliations:** a School of Public Health and Social Sciences, Muhimbili University of Health and Allied Sciences

## Abstract

**Background:**

Cervical cancer is the fourth most common cause of death among women of reproductive age (15-49 years). In Tanzania, cervical cancer is the first cause of mortality and morbidity among females with cancers. The disease impact is highly associated with a lack of adequate knowledge and a negative attitude toward cervical cancer screening among healthcare workers (HCWs).

This study aimed to assess knowledge and attitude toward cervical cancer screening among female students as future HCWs from allied health colleges in the Shinyanga region.

**Method:**

Descriptive cross-sectional study data was collected from 420 students in allied health colleges using a self-administered questionnaire. Data were analyzed using Statistical Package for Social Sciences (SPSS) version 22, Likert, and brooms cut-off points. The association between cervical cancer screening and the independent variables on knowledge, attitude, and other related factors was established by using logistic regression, and the Odds Ratio (OR) of greater than one, 95% confidence interval, and a *P*-value of <.05 was statistically significant.

**Results:**

Two-thirds of the respondents 276 (65.7%) had low knowledge, while only 34 (8.1%) had very good knowledge of cervical cancer and screening. Most of the respondents 298 (70.1%) had a favorable attitude toward cervical cancer screening. Only 52 (12.1%) had ever screened for cervical cancer. Logistic regression showed odds at 2.37 (95% CI, 1.30-4.31, *p=.005*) of taking the cervical screening test to students with the correct knowledge and positive attitude to cervical screening at 1.42 (95% CI, 0.32-6.29, *p=.647*)

**Conclusion:**

The study showed there is low knowledge of cervical cancer screening among female students in health and allied colleges, despite a favorable attitude toward the practice of screening. A well-integrated approach to providing comprehensive and practical aspects of cervical cancer screening during formal training in the curriculum for female students should be adopted to increase their knowledge and positive attitude toward screening.

## INTRODUCTION

Globally, cervical cancer is the fourth most common cause of death among females. The disease affects women of reproductive age and during their productive years, between 15-49 years.^1^ By 2020 the World Health Organization (WHO) reported an incidence of 604,000 cases of cervical cancer, with an expected rise to 863,000 in 2040. The annual death was expected to be 342,000 in 2020 and projected to be 539,000 by the year 2040.^1^

In the year 2020, the annual number of deaths due to cervical cancer was about 76,700 in Africa and it was projected to increase by 51% by 2040 if extensive measures are not implemented to combat the disease morbidity and mortality.^1^ In Tanzania, cervical cancer is the first cause of mortality among females with cancers. There are 10,200 incidences of cervical cancer with a morbidity and mortality rate of 6,530 cases and its incidence is projected to reach 21,600 in 2040 with a mortality of 14,100 due to a rapid increase in population.^1,2^ The most efficient way of cervical cancer prevention is the vaccination of 9 to 14-year-old girls and cervical cancer screening among women of reproductive age. Since the introduction of cervical cancer screening, there has been a reduction in incidence by 60%.^1,8^ The developed countries have achieved a coverage of 80% screening which prevents almost 90% of the disease.^2^

In Tanzania, the level of screening remains low at just 3.2% among women aged 18-69 years in comparison to 70% in high-income countries.^18^ This gap of low cervical cancer screening is highly attributed to inadequate knowledge and unfavorable attitudes toward the practice of cervical cancer screening among women. The gap in accessing cervical cancer screening is attributed to poor health-seeking behavior in developing countries as compared to developed countries^3,4^. This problem is further compounded by inadequate knowledge among healthcare workers (HCWs) on screening and poor attitudes toward cervical cancer screening which consequently affect the efforts in cervical cancer prevention.^5^ HCWs are identified as the major source of information about cervical cancer screening and have a massive influence in sharing it which eventually increases behavior change and health seeking on cervical cancer screening among women.^6,7^ Through understanding cervical cancer and methods of screening, women can have a wider knowledge of the disease, and its risk factors and understand how it can be prevented.

The study focused on female students only from allied health colleges as future HCWs, as they comprise a group that is eligible for screening for cervical cancer. Additionally, they are a primary source of health information to the patients on the disease and its prevention and hence play a crucial role in cervical cancer prevention. Also, if these students have adequate knowledge and a good attitude, they will act as change agents in the community on cervical cancer screening and once they graduate, they can reach the wider community and provide education on the importance of screening. However, little is known regarding their level of knowledge and attitude toward cervical cancer screening. The study, therefore, aimed to assess these attributes among female students in allied health colleges in the Shinyanga region.

## METHODS

This quantitative, descriptive cross-sectional study was conducted among female students in allied health colleges in the Shinyanga region between July and September 2022. Shinyanga region has three allied health colleges, and a total of 800 female students were admitted to the colleges during the time the study was conducted.

### Sample Size

The sample size of 420 was obtained using the Kish Leslie formula (1965) for cross-sectional studies, The assumption of the knowledge and attitude of cervical cancer screening among college students of 50% was extracted from a study by Obol.^8^ and used in the calculation of the study sample size.

N = Z^2^PQ/e^2^

Where;

N= minimum sample size

Z= standard normal deviation, set at 1.96 which corresponds to a 95% confidence interval.

P = is the assumption on knowledge and attitude of cervical cancer screening among college students of 50%

Q = (1 – p) = (1 – 0.5) = 0.5

E = margin error at 5% (standard value of 0.05).

N = ×0.5×0.5/0.05^2^

N=384

Adding a non-response rate of 10%, the minimum sample size for this study was around 420.

### Sampling Technique

A simple random sampling method was used to select respondents and to ensure that all female students had an equal chance to participate in the study. A proportionate sampling was used to identify participants from the three allied health colleges.

Out of the 420 respondents who were recruited in this study, 260 were from Kolandoto College of Health and Allied Sciences, 80 were from Kahama College of Nursing, and another 80 participants were from Kahama College of Health and Allied Sciences. The variation in the number of participants from the institutions was based on different sample fractions (420/800 = 0.525) and the number of admissions in each college.

Kolandoto College of Health and Allied Sciences had a sample fraction of 0.52 (260/500) participants from each year including first year 63, second year 78, and third year 120. The first year whose sample fraction was 0.57 included 20 respondents from the College of Nursing and 43 from the College of Clinical Medicine. Second-year participants whose sample fraction was 0.52 included 36 respondents from the College of Clinical Medicine and 42 from the College of Nursing. Participants from year three whose sampling fraction was 0.48, included 48 respondents from the College of Nursing and 72 from the College of Clinical Medicine.

From Kahama College of Health and Allied Sciences with a sample fraction of 0.53 (80/150) a total of 80 participants were involved: 18 from the first year, second year 25, and third year 37. Furthermore, from the first-year participants with a sample fraction of 0.45, seven respondents were from the College of Nursing and 11 from the College of Clinical Medicine. For year two participants whose sample fraction was 0.48, 11 respondents were recruited from the College of Nursing and 14 from the College of Clinical Medicine. Likewise, for the third-year participants with a sample fraction of 0.53, 15 respondents came from the College of Nursing and 22 from the College of Clinical Medicine.

For Kahama College of Nursing with a sampling fraction of 0.53 (80/150), a total of 80 participants were included, 18 from the first year, 25 and 37 from the second and third year respectively

### Data Collection

Data was collected by using a self-administered questionnaire which consisted of four sections: Section 1 socio-demographic characteristics of the respondents; Section 2 addressed the knowledge toward cervical cancer screening whereas Section 3 addressed the attitude toward cervical cancer screening. Section 4 addressed factors related to engagement in cervical cancer screening and focused on the identification of key factors that affect the screening. The research questionnaire was pretested on 20 female students from Kigoma College of Health and Allied Sciences, which was not involved in the study. The questionnaire was reviewed based on responses from the participants and adjusted to ensure that it measured the objectives of the study. The filled questionnaires were stored in privacy and were not included in the main study.

### Data Analysis

Data were checked for completeness, cleaned, and subjected to the analysis process using Statistical Package for Social Sciences (SPSS) version 22. The data were then summarized by using percentages and frequency for categorical data, mean and standard deviation for continuous variables, and presented in tables and charts. Responses were limited to “Yes,” “No,” and “I do not know” in measuring the knowledge on cervical cancer screening. Knowledge scores were computed using Broom's cut-off points which use three hierarchical models to classify the educational learning objective based on the level of complexity and specificity. In this study, the Brooms cut of points of good, moderate, and low were categorized as follows; where a participant correctly responded to at least 8-10 questions (equivalent to 80-100%), the level of knowledge was categorized as good, whereas participants who correctly scored 6-7 questions (60-70%) were classified as having moderate level of knowledge. Any respondent correctly responding to five questions and below was scored as having a low level of knowledge.

A Likert scale was used to measure the attitudes toward cervical cancer screening. The attitudinal statements had a scale of 5-strongly agree, 4-agree, 3-Neutral, 2-Disagree, and 1-Strongly disagree. Out of all statements, if a participant showed a positive attitude (agree and strongly agree) to any of the five statements she was considered to have a positive attitude toward screening.

The relationship between dependent and independent variables was analyzed by using logistic regression and the Odds Ratio (OR) of greater than one, 95% confidence interval, and a p-value of <0.05 was statistically significant.

### Ethical Issues

The study was granted ethical clearance from the Muhimbili University of Health and Allied Sciences (MUHAS) Institutional Review Board, Ref.No.DA.282/298/01.C/. Permission to collect data from the Allied Health Colleges in Shinyanga was requested from the respective Principals in each of the colleges. Additionally, all selected students were informed of the voluntary nature of participation in the study and of their freedom to withdraw from the study at any time without being penalized or facing any consequences. The process of data collection observed all principles of confidentiality, and all participants were asked to sign a consent form before being given the study questionnaire to fill out.

## RESULTS

### Social and Demographic Characteristics.

Most of the respondents 335(79%) were aged 17 to 24 years. Their age range was 17-34 years, and the mean age was 22.41, SD 2.949. Regarding marital status, the highest proportion of the respondents 355(84.2%) reported being single at the time of data collection while only about a tenth of them 40(9.5%) reported being married. The largest proportion of the respondents was in their third year of studies, 198(47.1%) whereas the least proportion was in their first year 99(23.6%) (Table 1).

**TABLE 1:** Socio-demographic Characteristics of the Study Respondents

Characteristic	Number	Percentag (%)
Age group (years)		
17–24	335	79.0
25–29	76	19.0
30–34	9	2.0
Marital status		
Single	355	84.5
Married	40	9.5
Cohabiting	22	5.2
Separated	3	0.7
Year of Study		
First-year	99	23.6
Second year	123	29.3
Third year	198	47.1

### The Proportion of Respondents who had ever Screened for Cervical Cancer

The study findings revealed that among the 420 female students in health colleges who participated in the study, only 52(12.1 %) had ever been screened for cervical cancer.

### Knowledge of Cervical Cancer among Female Students in Health Colleges

With regards to knowledge of the causative and risk factors of cervical cancer; most students 372(88.6%) attributed cervical cancer to a virus. As for risk factors for the cancer of the cervix, the highest proportion of the respondents 349(83.1%) correctly mentioned having multiple sexual partners, followed by 326(77.6%) who mentioned early sexual intercourse and a further 321(76.4%) who mentioned human papillomavirus. However, about a quarter of the students 108(25.7%) did not implicate cigarette smoking as a risk factor for cervical cancer.

With regards to symptoms of cancer of the cervix, most respondents 320(76.2%) correctly mentioned irregular vaginal bleeding, followed by foul-smelling vaginal discharge, 308(73.3%) whereas 287(68.3) mentioned postcoital bleeding. Concerning knowledge on the prevention of cervical cancer, most of the participants 368(87.6%) responded to avoiding multiple sexual partners followed by 338(80.5%) who mentioned avoiding early sexual intercourse and a further 324(77.2%) who said getting the HPV vaccine.

Visual inspection of the cervix (305, 72.6%) and human papillomavirus DNA testing 286 (68.1%) were the two most common cervical cancer screening methods known to the students while the least known method was liquid-based cytology, 195(46.4%). (Table 2).

**TABLE 2:** Knowledge of Cervical Cancer Among Female Students in Allied Health Sciences Colleges in Shinyanga (N=420)

Variable	Yes	
	Number	%
The causative agent of cervical cancer		
Virus	372	88.6
Bacteria	109	26.0
Fungi	67	16.0
Parasite	39	9.3
Risks factors for cancer of the cervix		
Having multiple sexual partners	349	83.1
Human papilloma virus	321	76.4
Early sexual intercourse	326	77.6
Cigarette smoking	244	58.1
Symptoms of the cancer of the cervix		
Vagina foul-smelling discharge	308	73.3
Vagina irregular bleeding	320	76.2
Post-coital bleeding	287	68.3
Prevent getting cancer of the cervix		
Avoid multiple sexual partners	368	87.6
Avoid early sexual intercourse	338	80.5
Get HPV vaccinations	324	77.2
Quit cigarette smoking	265	63.1
Screening for signs of the diseases	330	78.8
Whom to screen for cancer of the cervix		
Women of 25 years and more	323	77.1
Prostitutes	245	58.3
Women who do not have a child above 35 years	238	56.8
Methods used for screening of cervical cancer		
Pap smear	246	58.8
Visual inspection of the cervix	305	72.6
Human papillomavirus DNA testing	286	68.1
Liquid-based cytology	195	46.4
Often a woman repeats a screening test		
Once a year	105	59.0
Every 3 years	77	54.6
Every 5 years	38	33.9
Cervical cancer screening procedure should be included in routine tests	328	78.1

### Overall Knowledge of Cervical Cancer among Female Students in Health and Allied Colleges

The study summarized the overall knowledge according to Bloom's cut-off points and shows that 276(65.71%) respondents had low knowledge of cervical cancer and CI of 65.71% is within the range (=61.03-70.0), 110(26.19) had moderate knowledge (95% with CI of 26.1% and within the range in (22.20-30.62), and only 34(8.10) had excellent knowledge in cervical cancer with (95% CI of (5.83-11.11). With all variables showing the CI within their respective 95% confidence interval, it shows the findings are statistically significant to establish the association between the knowledge and the uptake of cervical cancer (Table 3).

**TABLE 3:** Overall Knowledge of Cervical Cancer According to Bloom's Cut-Off Points

Variable	n (%)	95% Confidence Interval
Low knowledge	276 (65.71)	61.03–70.11
Moderate knowledge	110 (26.19)	22.20–30.62
Excellent knowledge	34 (8.10)	5.83–11.11

### Attitude on cervical cancer screening among respondents

The attitude towards cervical cancer screening among the respondents revealed that the key attitude statements with the highest score included, “it is of medical importance to detect cervical cancer early with a mean score of 4.71 (SD = 0.621), followed by “as a health care worker to be, I feel responsible to address the importance of cervical cancer screening to all women” with a mean score of 4.65 (SD = 0.721). The statement “I prefer my cervical cancer screening to be conducted by a professional woman rather than a man” had the lowest subscription with a mean score of 4.07(SD=1.114) (Table 4).

**TABLE 4:** Attitude on Cervical Cancer Screening Among Respondents

Statement	Likert Scale (1–5)					Mean	
	SD n (%)	DA n (%)	NE n (%)	AG n (%)	SA n (%)	Score (1–5)	SD
It is of medical importance to detect cervical cancer early.	-	11 (2.6)	5 (1.2)	77 (18.3)	327 (77.9)	4.71	0.621
As a woman, you are at risk of getting cervical cancer.	13 (3.1)	20 (4.8)	22 (5.2)	125 (29.8)	240 (57.1)	4.33	0.993
There are effective methods for the prevention of cervical cancer.	2 (0.48)	18 (4.3)	39 (9.3)	132 (31.4)	229 (54.5)	4.35	0.854
Cancer of the cervix can be treated when early diagnosed.	2 (0.5)	11 (2.6)	19 (4.5)	117 (27.9)	271 (64.5)	4.53	0.745
Screening helps in the prevention of cervical cancer.	3 (0.7)	15 (3.6)	28 (6.7)	132 (31.4)	242 (57.6)	4.42	0.823
Screening helps in the identification of cervical cancer in treatable stages.	4 (1.0)	9 (2.1)	19 (4.5)	153 (36.4)	235 (55.9)	4.43	0.792
As a woman, I am willing to participate in the screening for cervical cancer.	5 (1.2)	22 (5.2)	31 (7.4)	113 (26.9)	248 (59.1)	4.37	0.941
I prefer my cervical cancer screening to be conducted by a professional woman.	14 (3.3)	39 (9.3)	44 (10.5)	127 (30.2)	196 (46.7)	4.07	1.114
As a healthcare worker to be, I feel responsible to address the importance of cervical cancer screening to all women.	4 (1.0)	9 (2.1)	10 (2.4)	84 (20.0)	313 (74.5)	4.65	0.721

SA=strongly agree, AG=agree, NE=neutral, DA=disagree, SD=strongly disagree

### Overall attitude toward cervical cancer according to Bloom's cut-off points

Findings show that 298(70.95%) respondents had a positive attitude toward cervical cancer screening with (95% CI ranging between 66.4 and 75.11 (Table 5). These findings are significant in establishing the association between the attitude and uptake of cervical cancer screening with the 95% CI in the variable on neutral attitude 23.33 (19.52-27.63) and negative attitude 5.71 (3.85-8.39) which is also statistically significant.

**TABLE 5:** Overall Attitude on Cervical Cancer According to Bloom's Cut-Off Points

Variable	n (%)	95% Confidence Interval
Altitude		
Negative attitude	24 (5.71)	3.85–8.39
Neutral attitude	98 (23.33)	19.52–27.63
Positive attitude	298 (70.95)	66.4–75.11

### Overall attitude toward cervical cancer according to Bloom's cut-off points

Findings show that 298(70.95%) respondents had a positive attitude toward cervical cancer screening with (95% CI ranging between 66.4 and 75.11 (Table 5). These findings are significant in establishing the association between the attitude and uptake of cervical cancer screening with the 95% CI in the variable on neutral attitude 23.33 (19.52-27.63) and negative attitude 5.71 (3.85-8.39) which is also statistically significant.

### Other factors affecting the uptake of cervical cancer screening among respondents.

The study revealed that 59.1% (95% CI = 53.9% - 64.1%) affirmed the inadequacy of information about cervical cancer screening, while 50.1% (95% CI = 45.1%-55.4%) affirmed for lack of individual willingness to participate in the uptake of cervical cancer screening test (Table 6).

**TABLE 6:** Factors Related to the Uptake of Cervical Cancer Screening Among Female College Students in the Shinyanga Region Who Did Not Screen (N = 368)

Variable	Not Screened	
	Number	%
Inadequate information about cervical cancer screening	218	59.1
Low-risk perception	131	35.5
Lack of individual willingness	185	50.1
Fear of positive results	151	40.9
Fear of screening procedures	151	40.9
Fear of pain during treatment	134	36.3
Feeling embarrassed when examined by a male provider	143	38.8
Peer influence to participate in cervical cancer screening	192	45.7

### Association between uptake of cervical cancer screening and knowledge

The study shows that respondents with the correct knowledge of the risk factors for cancer of the cervix had two times the odds of screening for the disease 2.37 (95% CI, 1.30-4.31, p=0.005). Similarly, respondents who were knowledgeable about the symptoms of cervical cancer had two times the odds of screening at 2.17 (95% CI, 1.19-3.98, p=0.012) (Table 7).

**TABLE 7:** Association Between Uptake of Cervical Cancer Screening and Knowledge Among Female College Students in Shinyanga

Knowledge of cervical cancer	Screened for cervical cancer		Bivariate	Multivariate
	Yes, n (%)	No, n (%)	COR, 95% CI, *p-value*	CoR, 95% CI, *p-value*
Causative agent				
Not correct	-	48 (100)	1	1
Correct	51 (13.7)	321 (86.3)	-	-
Risks factors				
Not correct	20 (8.2)	223 (91.8)	1	1
Correct	31 (17.5)	146 (82.5)	2.37, 1.30–4.31, 0.005	1.48, 0.71–3.08, 0.300
Symptoms				
Not correct	19 (8.4)	208 (91.6)	1	1
Correct	32 (16.6)	161 (83.4)	2.17, 1.19–3.98, 0.012	1.43, 0.72–2.84, 0.301
Prevention				
Not correct	23 (9.5)	220 (90.5)	1	1
Correct	28 (15.8)	149 (84.2)	1.80, 0.99–3.24, 0.051	1.04, 0.51–2.14, 0.895
Who to be screened				
Not correct	26 (9.8)	239 (90.2)	1	1
Correct	25 (16.1)	130 (83.9)	1.77, 0.98–3.186, 0.058	1.31, 0.69–2.48, 0.409
Methods used for screening				
Not correct	32 (10.3)	279 (89.7)	1	1
Correct	19 (17.4)	90 (82.6)	1.84, 0.99–3.41, 0.052	1.31, 0.67–2.53, 0.427
When to repeat screening test				
Not correct	11 (12.0)	81 (88.0)	1	1
Correct	40 (12.2)	288 (87.8)	1.02, 0.50–2.08, 0.951	0.70, 0.32–1.55, 0.386
Inclusion of screening procedure in routine tests			
Not correct	13 (10.9)	106 (89.1)	1	1
Correct	38 (12.6)	263 (87.4)	1.18, 0.60–2.20, 0.631	0.89, 0.42–1.87, 0.759
Awareness about free screening program in Tanzania			
Not correct	50 (12.2)	359 (87.8)	1	1
Correct	1 (9.1)	10 (90.9)	0.72, 0.09–5.73, 0.755	0.68, 0.08–5.61, 0.720

### Logistic regression of the association between cervical cancer screening uptake and knowledge and attitude on cervical cancer

Results revealed that students with moderate knowledge of cervical cancer had significantly higher odds of taking the cervical screening test, 3.05 (95% CI, 1.62-5.75, p=0.001) compared to other knowledge levels. In the multivariate analysis, the odds increased to 3.28 (95% CI, 1.71-6.25, p=0.001). Likewise, students with neutral attitudes had higher (but not statistically significant) odds of taking the cervical screening test, 1.99 (95% CI, 0.42-9.35, p=0.384) compared to their colleagues with negative and positive attitudes. The insignificant odds remain the same during multivariate analysis 1.99 (95% CI, 0.41-9.62, p=0.390). (Table 8)

**TABLE 8:** Logistic Regression Association Between Knowledge and Attitude and Cervical Cancer Screening Uptake

Variable	Cervical cancer screening		Bivariate	Multivariate
	Yes, n (%)	No, n (%)	COR, 95% CI, *p-value*	COR, 95% CI, *p-value*
Knowledge level				
Low	22 (8.0)	254 (92.0)	1	1
Moderate	23 (20.9)	87 (79.1)	3.05, 1.62–5.75, 0.001	3.28, 1.71–6.25, 0.001
Excellent	6 (17.7))	28 (82.3)	2.47, 0.93–6.61, 0.071	2.67, 0.98–7.24, 0.054
Attitude				
Negative	2 (8.3)	22 (91.7)	1	1
Neutral	15 (15.3)	83 (84.7)	1.99, 0.42–9.35, 0.384	1.99, 0.41–9.62, 0.390
Positive	34 (11.4)	264 (88.6)	1.42, 0.32–6.29, 0.647	1.15, 0.25–5.26, 0.856

## DISCUSSION

This study aimed to assess knowledge, attitude, and other factors associated with the uptake of cervical cancer screening among female students in allied health colleges in the Shinyanga region.

### The Proportion of Screening Among Female Students

The study interestingly revealed that only 52(12.1%) of female students in allied health colleges have ever screened for cancer of the cervix. Although this proportion is low, it is however higher when comparing the screening rate in this study with other studies and the respondents showed good knowledge and favorable attitudes toward screening. A study in Nigeria revealed that only 4.7% of female students reported having been screened.^9^ Another related study in Eastern Ethiopia showed only 17 respondents out of 730(2.5%) have ever been screened despite 60% of them having a good attitude towards screening.^10^ Findings from other studies in Kilimanjaro Tanzania showed that only 31(9.1%) respondents had screened^11^ whereas, in Southern Eastern Nigeria, only 27(7.2%) had at least one screening in their lifetime.^12^ Contrary to the current study, a high proportion of screenings among students has been reported in Botswana among university female students which showed that 92 out of 335(27.5%) participants had ever done cervical cancer screening.^4^ A study conducted in Benin in 2019 on awareness and uptake of cervical cancer screening among female students in a College of basic science at the University of Benin revealed that 36/200(18%) of all respondents had ever had a cervical cancer screening.^13^ The practice of cervical cancer is very low even though most participants in the reported studies had a good knowledge and attitude on cervical cancer prevention.

The study highlights the fact that these female students in allied health colleges require a well-integrated mechanism (program/projects) for them to actively engage in cervical cancer screening programs which will eventually support their positive responses toward screening and practice to themselves and all females they encounter.

### Knowledge of Cervical Cancer Screening Among Female Students

In this study, respondents showed low knowledge (65.7%) of cervical cancer screening. Unlike this study, a survey study conducted in Kilimanjaro on knowledge and uptake of cervical cancer screening among university students revealed a lower proportion of 126(39.1%) respondents with poor knowledge.^11^ Different reasons contributed to poor knowledge including lack of adequate information about the disease including causative agents, risk factors and preventions, not being at risk of getting the disease, and not being aware of cervical cancer screening programs. These results are quite different compared to other studies conducted in Botswana^4^, Northern Uganda^8^, Cote d Ivoire^14^, Nigeria^9^, and Iran.^15^ These studies showed there is a good knowledge of cervical cancer screening among female students in universities and nursing colleges when compared to this study. The low level of knowledge among female students from this present study is very alarming because these students have an opportunity to formal education on cervical cancer prevention in their curriculum and most of them were in their final year of studies. Their low level of knowledge might later affect their active engagement in prevention activities.

### Attitude Towards Cervical Cancer Among Female Students

This study has shown that there is a very good attitude toward cervical cancer screening. About 70% of the students have shown a good attitude toward cervical cancer screening. Of the 317 (87.6%) who did not take up the screening test are willing to participate in screening activities and 350(88.2%) also believe as future healthcare providers they feel responsible for addressing the importance of cervical cancer screening to all women. This study has shown similarities in the attitude aspect when compared with other studies conducted at Gondar University with 67.7% favorable attitude^16^, in Iran 90% showed a good attitude win encouraging a family member to take up the screening test.^15^. A positive attitude toward screening has also been shown by a study conducted in Southern Eastern Nigeria in 2021 on determinants of cervical cancer uptake among female students in a tertiary institution. In the study, 93% (349 respondents) had a good attitude toward screening.^12^ All these studies show good attitudes toward cervical cancer among students, highlighting the willingness and positive engagement of female students toward cervical cancer screening.

### Factors Associated with Cervical Cancer Screening

The study identified different factors affecting female students' practices toward cervical cancer screening including inadequate information about cervical cancer, peer influence on cervical cancer screening, fear of the screening procedure, and feeling embarrassed when examined by male healthcare providers. Such factors were also documented in a study in Bahrain and ^17^ in Jordan. Factors like fear of negative results, the procedure itself, the instrument, the expectation of pain, or bleeding, embarrassment, a fatalistic belief regarding screening, lack of time, husband's disapproval, the attitude of the healthcare provider, and the absence of female physicians ^18^ have also been documented.

Lack of individual willingness to be screened 185(50.1%) was also a reason which was identified by a study conducted in Eastern Ethiopia^10^ and a study in Kilimanjaro 114(30.4%).^11^

When all these factors are well considered during the planning and implementation of future cervical cancer screening strategies, they will highly increase the active engagement of the female population in screening activities.

### Study Limitations and Mitigations

Some of the information collected was sensitive which might have affected the participant's ability to provide correct responses without fear of an invasion of privacy. The study respondents were assured that all information collected was stored safely and confidentially. All participants were assigned IDs and no names were involved or recorded anywhere hence their identity was unknown. Also, the study was a cross-sectional design which cannot establish the causal relationship between the independent and dependent variables.

## CONCLUSION

The study showed there is low knowledge of cervical cancer screening among female students. Although, the majority showed to have a favorable attitude toward cervical cancer screening as a method to prevent the disease, still only a minority of the correspondents had ever had cervical cancer screening. This affects their contribution as future healthcare workers in providing adequate information to women on cervical cancer screening as well as deterring them from convincing other girls and women to participate in the screening which will ultimately reduce cervical cancer morbidity and mortality.

### Recommendations

- To increase students' knowledge and attitude toward cervical cancer screening, there should be the active involvement of students in strengthening their knowledge on cervical cancer screening through the provision of information education and communication through training, mentorship, seminars on cervical cancer prevention through clubs and use of different occasions. like graduation, training, and other public gathering to spread the importance of screening for cervical cancer.

- There should be the active involvement of stakeholders including students in ensuring that all those programs which will be introduced to increase their knowledge and uptake of cervical cancer are being sustained through their active participation in clubs, mentorship, and Cervical awareness month in February. Administrations to ensure support of the existing program through funds in preparation and commemoration of cervical cancer activities in respective colleges, and training governing boards including NACT-VET through revising and developing sustainable education curriculum focusing on increasing cervical cancer knowledge and screening uptake in all allied health colleges which will have an impact to female students themselves and women they will provide services in their lifetime.

- The study has established the backbone of knowledge and attitude toward cervical cancer screening among female students in allied health colleges, there is a need to conduct further studies to observe if the planned activities on raising the knowledge and uptake have an impact and the effectiveness of the strategies in ensuring students participation in cervical cancer screening which will reduce disease morbidity and mortality.
